# Passive smoking and stroke in men and women: a national population-based case-control study in China

**DOI:** 10.1038/srep45542

**Published:** 2017-03-31

**Authors:** Lei Hou, Wei Han, Jingmei Jiang, Boqi Liu, Yanping Wu, Xiaonong Zou, Fang Xue, Yuanli Chen, Biao Zhang, Haiyu Pang, Yuyan Wang, Zixing Wang, Yaoda Hu, Junyao Li

**Affiliations:** 1 Department of Epidemiology and Biostatistics, Institute of Basic Medical Sciences Chinese Academy of Medical Sciences/School of Basic Medicine Peking Union Medical College, Beijing, China; 2 National Center for Chronic and Noncommunicable Disease Control and Prevention, Chinese Center for Disease Control and Prevention, Beijing, China; 3 Cancer Institute & Hospital, Chinese Academy of Medical Sciences/Peking Union Medical College, Beijing, China

## Abstract

An association between passive smoking and stroke is unclear in China, particularly the association with hemorrhagic stroke. This study included 16205 deaths due to stroke aged ≥30 years and 16205 non-stroke controls randomly selected and frequency-matched to cases on gender and age. Smoking of spouses, defined as ≥1 cigarette per day for up to 1 year, was taken as a measure of exposure to passive smoking of subjects that was retrospectively ascertained by interviewing surviving spouses. After adjustment for variables, passive smoking increased the risk of death by 10% (odds ratio (OR), 1.10; 95% confidence interval (CI), 1.05–1.16) for all strokes, by 10% (OR, 1.10; 95% CI, 1.04–1.16) for hemorrhagic stroke, and by 12% (OR, 1.12; 95% CI, 1.03–1.23) for ischemic stroke, compared with non-exposure. This finding was highly consistent in men or women and in smokers or non-smokers, and was generally consistent among zones of China despite geographic diversity. The risk significantly increased with exposure-years and quantity of cigarettes smoked daily by spouses. This study indicated that passive smoking is associated with deaths from all-type strokes. It is highly advisable for the government to promote strong tobacco prevention and cessation programs and smoke-free environments.

The World Health Organization has estimated that 15 million people worldwide suffer a stroke every year. Of these, 5 million die and another 5 million are left permanently disabled. China has more than 300 million smokers and 740 million non-smokers exposed to second-hand smoke^1^. Currently, active cigarette smoking is a well-established major preventable risk factor for stroke, and 12.4% of incident strokes can be attributed to current smoking^2^. Passive exposure to tobacco smoke is associated with cancer and coronary heart disease, and may be responsible for 20% of all deaths in the working-age population of the Western countries such as the United Kingdom^3^,^4^. However, the relationship between passive smoking and strokes is unclear, especially in China that has seven million patients with stroke. An association between passive smoking and stroke was indicated in one cohort study for female non-smokers rather than male non-smokers^5^, as supported by some case-control studies and meta-analyses^6^,^7^,^8^. Nevertheless, another cohort study using baseline serum cotinine concentration as an indicator of passive smoking in male nonsmokers and other case-control studies did not support this association^9^,^10^,^11^,^12^. Furthermore, two other prospective cohorts provided a self-contradictory result in smokers and nonsmokers^13^,^14^. One study showed an increased risk associated with passive smoking in smokers rather than non-smokers as dramatically compared with non-smokers rather than smokers in the other study. More importantly, a study on association between passive smoking and hemorrhagic stroke was lacking, though the association with all strokes, in particular ischemic stroke, was observed in the previous studies^5^,^6^. In Oriental countries such as China, where stroke, particularly hemorrhagic stroke, is much more frequent compared with Western countries^15^, there are not enough data to support an association between passive smoking and stroke because of the non-significance of statistics^11^,^16^, cross-sectional design of studies^17^,^18^, inconsistent results between types of stroke^17^,^19^ or exposure place such as household and workplace within the same study^20^, or a population limited to women^17^,^18^,^19^,^20^.

The main objective of the current study was to investigate whether there was an association between exposure to passive smoke and death from all-type strokes in China.

## Results

The 16 205 cases included 12 579 with hemorrhagic stroke (97.8% intracerebral hemorrhage and 2.2% subarachnoid hemorrhage) and 3626 with ischemic stroke. Overall, the cases and 16 205 controls had similar characteristics on zones, ethnicity, education, and active smoking; however, there was a slightly higher prevalence of passive smoking of 30.8% (16.2% for men and 60.0% for women) in the case group compared with 29.1% (15.0% for men and 57.4% for women) in the control group (Table 1). This was associated with a slightly increased risk of deaths from all strokes by 8% (OR, 1.08; 95% CI, 1.03–1.14). After adjustment for age only, there was an increased risk of 9% (OR, 1.09; 95% CI, 1.02–1.18) in men and 12% (OR, 1.12; 95% CI, 1.04–1.21) in women. This risk in each age span of nonsmokers and smokers was shown in Table 2.

The fully adjusted models showed that exposure to passive smoking was associated with an increased risk of death by 10% (OR, 1.10; 95% CI, 1.05–1.16) for all stroke, by 10% (OR, 1.10; 95% CI, 1.04–1.16) for hemorrhagic stroke including subarachnoid hemorrhage (OR, 1.05; 95% CI, 0.79–1.40) and intracerebral hemorrhage (OR, 1.22; 95% CI, 1.17–1.28), and by 12% (OR, 1.12; 95% CI, 1.03–1.23) for ischemic stroke, compared with non-exposure. The increased risk was 14% (OR, 1.14; 95% CI, 1.06–1.22), 13% (OR, 1.13; 95% CI, 1.05–1.22), and 16% (OR, 1.16; 95% CI, 1.03–1.30), respectively, for non-smokers, and 7% (OR, 1.07; 95% CI, 0.99–1.17), 7% (OR, 1.07; 95% CI, 0.98–1.18), and 8% (OR, 1.08; 95% CI, 0.95–1.24), respectively, for smokers independent of years of active smoking. This finding was highly consistent in men or women (Table 3).

ORs were then calculated for different zones to clarify the geographic diversity of the associations. In general, the positive association between passive smoking and deaths from stroke was consistent between zones, although geographic diversity existed as shown in Table 4. For example, exposure to passive smoking was associated with an increased risk of death from hemorrhagic stroke by 6% (OR, 1.06; 95% CI, 0.94–1.20) and 5% (OR, 1.05; 95% CI, 0.95–1.16) in the northern and southern coastal China and 17% (OR, 1.17; 95% CI, 1.01–1.35) and 23% (OR, 1.23; 95% CI, 1.09–1.40) in the northern and southern inland China, respectively.

A dose-response relationship between exposure-years of passive smoking and stroke deaths was found (Table 5). For all strokes, ORs increased to 1.09 (95% CI, 0.94–1.26) for 1–19 years, 1.12 (95% CI, 1.04–1.20) for 20–39 years, and 1.11 (95% CI, 1.04–1.19) for ≥40 years of passive smoking (*p* for trend <0.001) after adjustment for all variables, compared with no exposure to passive smoke. The quantity of cigarettes smoked daily by spouses significantly increased the risk of death related to all strokes (*p* for trend <0.001). Similar associations were observed for hemorrhagic stroke and ischemic stroke. This finding was also consistent in populations with level 1 and level 2 of diagnosis.

Results from a sensitivity analysis based unmeasured or residential confounding were shown in Table 6. We found that failure to adjust for 5 potential confounders such as hypertension, high total cholesterol (TC), low high density lipoprotein cholesterol (HDL-C), high triacylglyceride (TG), and overweight would slightly change risk estimates by less than 1% compared with the “true” relative risk and this change was not substantial for estimates of spots and 95% CIs on all-type strokes. Due to some uncertainty on association of passive smoking with 5 confounders, we changed the OR from 1.01 assumed to 0.9 or 1.1 in this analysis, respectively, and the above finding was remained (data not shown).

## Discussion

To the best of our knowledge, this is the first study that indicates an overall association of passive smoking with death related to all strokes, hemorrhagic strokes, and ischemic strokes among both men and women. Based on the results, we found passive smoking increased the risk of stroke deaths in both smokers and non-smokers.

Hemorrhagic strokes account for 31%–64% of all strokes in oriental countries, such as China, as shown in the Monitoring Trends and Determinants in Cardiovascular Disease (MONICA) study. The proportion was up to 77.6% in our study. However, there are few studies on the association between passive smoking and hemorrhagic stroke. In an international population-based case-control study, passive smoking did not increase the risk of subarachnoid hemorrhage (OR, 0.9; 95% CI, 0.6–1.5)^10^, and there was a non-significant association with hemorrhagic stroke (OR, 1.10; 95% CI, 0.52–2.34) in a Chinese cross-sectional study^17^. Our study found a positive association between passive smoking and death from hemorrhagic strokes, with a significant dose-response effect. This was consistent with a Japanese cohort study in which passive smoking increased the risk of death from intracerebral hemorrhage by 35% (hazard ratio [HR], 1.35; 95% CI, 0.94–1.94) and from subarachnoid hemorrhage by 66% (HR, 1.66; 95% CI, 1.02–2.70) in women^19^. Although an association between passive smoking and ischemic strokes has been indicated in Western populations^21^, there has only been one cross-sectional study showing this association (OR, 1.56; 95% CI, 1.03–2.35) in Chinese subjects as opposed by the Japanese cohort study (HR, 0.95; 95% CI, 0.78–1.15)^19^. Our population-based case-control study added new evidence on the association for ischemic strokes and for all strokes in both men and women, different from many studies in either men or women. Also, the analysis on geographic variations of this study supported our findings above.

Previous studies mainly recruited non-smokers, but in our study both smokers and non-smokers were analyzed as independent subgroups. Currently, there were two studies that prospectively investigated the influence of passive smoking on stroke in smokers and nonsmokers. A finding from the First National Health and Nutrition Examination Survey Epidemiologic Follow-up Study showed that exposure to passive smoking significantly increased risk of all stroke (relative risk [RR], 5.7; 95% CI, 1.4–24) and ischemic stroke (RR, 4.8; 95% CI, 1.2–20) in smokers; however, this risk was not found in nonsmokers (RR, 0.9, 95% CI, 0.6–1.3 for all stroke; RR, 0.8, 95% CI, 0.6–1.3 for ischemic stroke) [13]. On the contrary, in the U.S.-based Health and Retirement Study, the risk of all strokes was increased by 42% (HR, 1.42; 95% CI, 1.02–1.92) in non-smokers compared with 3% (HR, 1.03; 95% CI, 0.75–1.49) in current smokers^14^. Our results indicated that passive smoking increased the risk of death from all-type strokes not only in non-smokers but also in smokers. This indicated that a smoke-free environment is both important for smokers and nonsmokers. A survey in hospitalized patients with coronary heart disease found that smokers with exposure to passive smoking at home had a lower likelihood of smoking cessation within 15 months than those without exposure (25.3% vs. 58.1%, *p* < 0.001); this suggested that passive smoking may hinder smoking cessation^3^.

Passive smoking could increase the risk of stroke through multiple mechanisms similar to active smoking. Carotid atherosclerosis has been associated with passive smoking^22^. Passive smoking was also found to be associated with elevated levels of C-reactive protein, homocysteine, fibrinogen, oxidized low-density lipoprotein cholesterol, and impaired endothelium-dependent arterial dilatation^23^. Passive smoking is the inhalation of environmental tobacco smoke composed of the smoke released by tobacco products and smoke exhaled by the smokers. Low-dose inhalation may trigger the above mechanisms, but stroke deaths associated with passive smoking could require an induction period, e.g. 20 years, as supported by the dose-response relationship in our study. Smoking cessation was also associated with a significant reduction of cardiovascular diseases; this may be related to a beneficial effect on metabolism such as a regularization of lipid profile^24^. For smoking quitters with cardiovascular diseases, occurrence of acute coronary syndrome and stroke could be reduced to values similar to nonsmokers between 5 and 15 years experiencing modern medical therapy^25^.

A key strength of the study was that it was the largest population-based case-control study that guaranteed consequent good generalizability, to our knowledge, on passive smoking in relation to stroke. As an underlying weak effect of passive smoking on strokes was indicated by previous studies, statistical power could be achieved by the large number of cases in the current study. Next, study subjects took part in a high-quality national survey in which 73.9% of cases and 71.7% of controls had reliable diagnoses from county-level or better hospitals and indicated a stronger dose-response relationship in relation to other subjects. In addition, this dose-response relationship was highly consistent for exposure types, such as time and quantity of exposure, and stroke types.

This study has some limitations. All available national mortality surveys or registry of China do not refer to information on physical examination such as blood pressure, blood lipid, and body mass index. Therefore, we had no these data included into our analysis. However, we removed all patients of both case and control groups with an underlying cause of death from cardiovascular diseases, in particular hypertension, other than stroke following the same standard. And a sensitivity analysis based unmeasured or residential confounding did not show these potential confounders would substantially change our results. Next, behaviors and doses of active smoking could be changed by diseases; however, this may be unlikely to change our results considering the consistency in each subgroup, exposure recalled over a long period before death, and highly fatal hemorrhagic strokes. Moreover, ex-smokers and exposure to second-hand smoke in the workplace could not be investigated because of a lack of relevant variables. Considering a low rate of quitting smoking (4.78%) and exposure in the public areas including workplace (5.74% vs. 26%–67% in the houses for non-smokers), according to the first National Smoking Survey of China conducted in 1984, and few studies indicating an association between exposure to smoking out of home and stroke^5^, our results could not be changed. Nevertheless, we might underestimate the association between passive smoking and strokes given the recall bias, a measurement error from surviving spouses, and misclassification due to diagnoses, particularly in population with diagnostic level 2 that likely had weaker dose-response relationship in relation to population with better diagnostic evidence.

In conclusion, this study across 28 provincial administrative regions of China provides the first evidence to support an association between passive smoking and death from all-type strokes, hemorrhagic or ischemic and both, among both men and women, in nonsmokers and smokers. Given the relationships between smoking and various forms of malignant tumors and cardiovascular diseases, and that a large population were exposed to second-hand smoking, it is highly advisable that the government develop strong tobacco prevention and cessation programs and compel provision of a smoke-free environment for all populations in China.

## Methods

A dataset from the China Nationwide Retrospective Mortality Survey, conducted from 1989 through 1991, was used. This survey included 1 136 686 all-cause deaths of subjects aged 30 years or older during the years 1986–1988 from 24 urban areas and 79 rural counties randomly chosen from over 2000 counties in China. These 103 areas with a population of 67 million were located in 28 out of 31 provincial administrative regions of China. Deaths were identified primarily from local administrative records and medical records. Over 500 interviewers usually worked in team of two in urban areas and four in rural areas. Each team included at least one trained clinical adjudicators. The underlying cause of each death was coded by trained nosologists from the Ministry of Health with experience of coding standard death certificates using the World Health Organization International Classification of Diseases, 9th revision (ICD-9).

**Figure 1:**
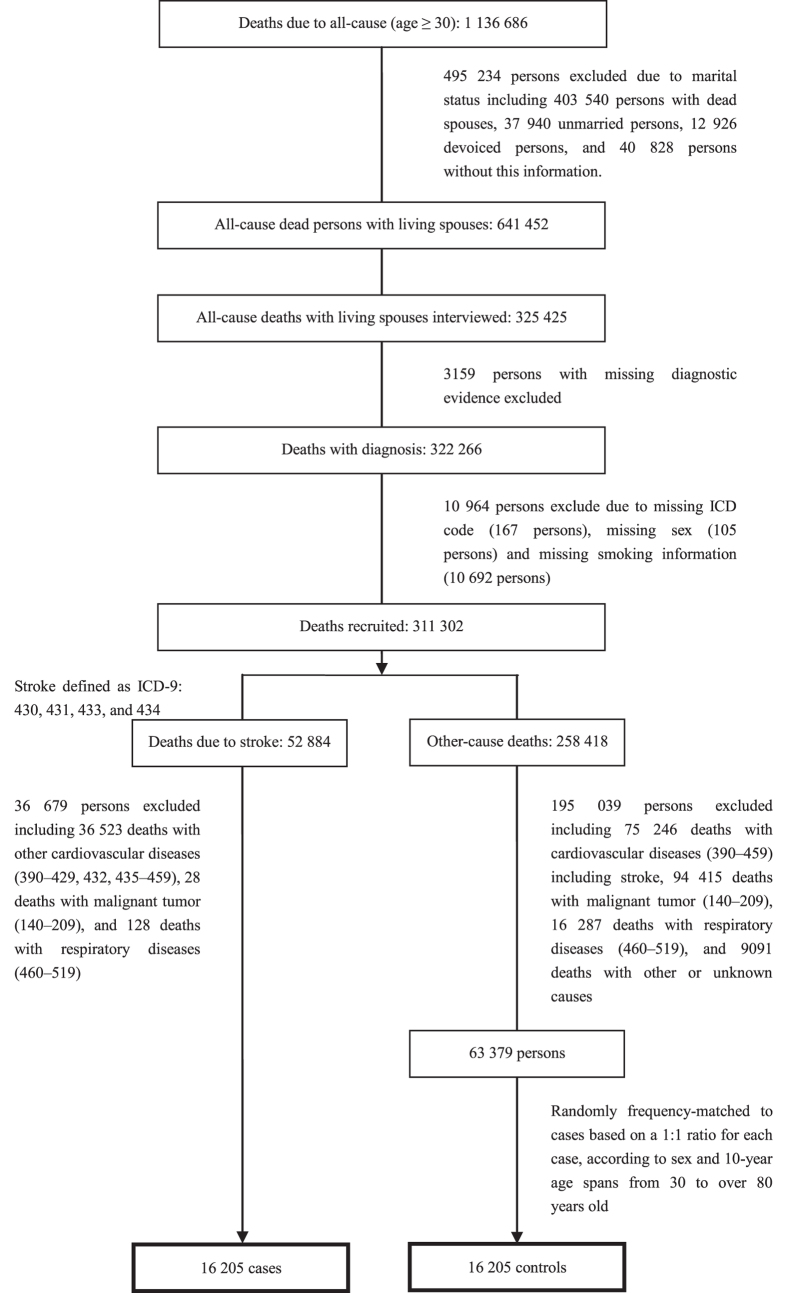
Flow chart illustrating the recruitment of cases and control.

The specific methods used to recruit all cases and controls are shown in Fig. 1. In the national mortality surveys and later mortality registry of China, diagnostic levels included autopsy, histological test, surgical operation, clinical assessment plus physical examinations and laboratory tests, clinical assessment, and deduction after death. Considering that clinical assessment was the most important diagnosis for stroke, e.g., “rapidly developing clinical signs of focal (or global) disturbance of cerebral function lasting more than 24 hours (unless interrupted by surgery or death) with no apparent cause other than a vascular origin” defined by the contemporaneous World Health Organization MONICA (monitoring trends and determinants in cardiovascular disease) definition^26^, all diagnostic levels of both cases and controls including deduction after death from trained staffs were employed in this study when recruiting stroke cases and non-stroke controls. Subjects with an underlying cause of death of stroke (430–431 for hemorrhagic strokes including subarachnoid hemorrhage and intracerebral hemorrhage, 433–434 for ischemic strokes including occlusion and stenosis of cerebral and precerebral arteries resulting in cerebral infarction, and 430–431 or 433–434 for all stroke) were included as cases. Those with other concomitant diseases related to smoking, such as other cardiovascular diseases (390–429, 432, 435–459) particularly including hypertension (401–405), malignant tumor (140–209.4), or respiratory diseases (460–519), were excluded. 0.3%, 0.8%, 0.5%, 57.8%, 36.6%, and 4.0% of stroke cases were based on the six diagnostic levels, respectively. For physical examinations and laboratory tests such as CT and lumbar puncture, there was no more detailed classification in the national survey. Following the same criteria for inclusion and exclusion as cases, non-stroke subjects were defined as controls. The control died from causes unrelated to smoking such as infectious and parasitic diseases (14.7%), endocrine, metabolic, and nutritional diseases (6.4%), blood and blood-forming organ diseases (0.9%), mental disorders (2.9%), nervous system diseases (4.4%), digestive system diseases (27.8%), genitourinary system diseases (9.5%), obstetric diseases (0.8%), musculoskeletal and connective tissue diseases (0.9%), and injury or poisoning (31.8%). Controls were randomly frequency-matched to cases based on a 1:1 ratio for each case, according to gender and 10-year age spans from 30 to over 80 years old. Finally, a total of 16 205 cases and 16 205 controls, from the northern coastal China, the northern inland China, the southern coastal China, and the southern inland China according to a traditional classification of zones (see our previous publication^27^), were included in the analysis. We also defined two diagnostic levels, the level 1 based on autopsy, histological test, surgical operation, or clinical assessment, including imaging or substantial laboratory tests in secondary or tertiary hospitals and the level 2 based on other diagnostic evidence. 73.9% of cases and 71.7% of controls had the level 1 of diagnoses.

During the survey from 1989 to 1991, surviving spouses of all deceased persons including cases and controls were interviewed to obtain information on smoking history according to the address provided by the administrative records. The interviewees described the smoking habits of their deceased family members and of themselves. These data were used to determine whether people had ever smoked by 1980, a period of time before onset of their disease, to minimize effects of behavior changes after the diagnosis of disease. Smoking of subjects and spouses was defined as at least 1 cigarette per day for up to 1 year. Smoking of spouses was taken as a measure of exposure to passive smoking of subjects. We used 20 years for the cut-off age for beginning smoking as the cut-off to define exposure-years of passive smoking; 20 years was likely the popularly acceptable age for marriage before the foundation of the People’s Republic of China (1949). For spouses aged 20 years or older at commencement of smoking, we defined years of passive smoking as the age at death for the case or control minus age at onset of daily smoking; for spouses who began smoking before the age of 20 years, the number of smoking years before this age was further subtracted. The number of cigarettes smoked per day was also recorded. All methods were carried out in accordance with relevant guidelines and regulations and all experimental protocols were approved by Chinese Academy of Medical Sciences. An oral informed consent was obtained from all subjects in this study. Additional details on this survey have been described elsewhere^28^,^29^.

Odds ratios (ORs) with 95% confidence intervals (CIs) were calculated using a non-conditional logistic regression model for estimating the effects of exposure to passive smoking on the risk of death from stroke and conducting a trend test^30^,^31^,^32^. The initial models were adjusted for age only in each subgroup such as gender, smoking status, and age spans. The fully adjusted models further included variables that could be associated with both exposure and outcomes, including gender, areas of residence, ethnicity, education, and active smoking (years of active smoking for smokers).

A sensitivity analysis based unmeasured or residential confounders was conducted. We used a method provided by Schneeweiss *et al*. to calculate percent bias from assumed unmeasured or residual confounders such as hypertension, high LDL-C, low HDL-C, high TG, and overweight^33^. Estimates of the confounder-disease associations were used according to high-quality oriental studies such as the Sino-MONICA and the Oyabe Study and prevalence of these confounders followed related Chinese guidelines^34^,^35^,^36^. Currently, relationship between passive smoking and the above confounders remained uncertain and we assumed this OR value as 1.01. Prevalence of passive smoking was defined as 0.30 from our study population.

All analyses were performed using the SAS 9.2 statistical software package. All *p*-values were two-sided except *p* trend tests, in which one-sided *p*-values were used. A *p*-value < 0.05 was considered statistically significant.

## Additional Information

**How to cite this article**: Hou, L. *et al*. Passive smoking and stroke in men and women: a national population-based case-control study in China. *Sci. Rep.* **7**, 45542; doi: 10.1038/srep45542 (2017).

**Publisher's note:** Springer Nature remains neutral with regard to jurisdictional claims in published maps and institutional affiliations.

## Figures and Tables

**Table 1:** Characteristics of case and control groups.

	Cases (n = 16 205)	Controls (n = 16 205)
Men (%)	66.7	66.7
Age (years)	64.9 ± 10.4	64.6 ± 10.7
Area (%)		
Foreland zone in north China	23.2	22.9
Foreland zone in south China	33.8	36.1
Inland zone in north China	17.8	16.6
Inland zone in south China	25.3	24.4
Han ethnicity (%)	95.4	95.2
Education (%)		
College or higher	2.5	1.4
Middle and high school	38.8	42.0
Primary school	38.1	36.5
Illiteracy	20.6	20.0
Active smoking (%)	47.3	46.5
Spouse smoking (%)	30.8	29.1

**Table 2:** Relationship between passive smoking and stroke death in subgroups of gender and age (years).

Age (years)	Men			Women			
	Numbers (percents of spouse smoking)		Age-adjusted ORs	Numbers (percents of spouse smoking)		Age-adjusted ORs	
	Cases	Controls	(95% CI)	Cases	Controls	(95% CI)	
Nonsmokers							
30~39	67 (9.0)	58 (3.5)	2.84 (0.55–14.73)	118 (69.5)	117 (65.8)	1.25 (0.72–2.18)	
40~49	182 (3.9)	182 (3.3)	1.22 (0.40–3.73)	430 (62.8)	446 (60.1)	1.12 (0.85–1.48)	
50~59	667 (10.0)	749 (7.2)	1.37 (0.94–1.99)	1242 (62.9)	1256 (61.6)	1.06 (0.90–1.25)	
60~69	1280 (14.0)	1365 (10.8)	1.32 (1.05–1.67)	1559 (58.8)	1538 (54.5)	1.21 (1.05–1.39)	
70~79	1365 (14.1)	1308 (14.1)	1.01 (0.81–1.26)	1042 (52.3)	1031 (51.6)	1.04 (0.87–1.23)	
80~98	396 (17.2)	421 (11.9)	1.54 (1.04–2.29)	197 (40.6)	202 (44.6)	0.86 (0.58–1.28)	
Smokers							
30~39	75 (2.7)	84 (3.6)	0.98 (0.15–6.28)	5 (40.0)	6 (66.7)	0.31 (0.03–3.87)	
40~49	288 (10.1)	288 (5.9)	1.65 (0.88–3.10)	49 (75.5)	33 (60.6)	1.93 (0.73–5.10)	
50~59	1304 (14.4)	1222 (13.2)	1.07 (0.85–1.35)	210 (76.2)	196 (67.9)	1.55 (1.00–2.41)	
60~69	2660 (18.7)	2575 (17.7)	1.07 (0.93–1.23)	310 (69.7)	331 (67.4)	1.13 (0.81–1.58)	
70~79	2102 (20.9)	2159 (21.2)	0.98 (0.85–1.14)	189 (61.9)	200 (60.5)	1.06 (0.71–1.60)	
80~98	430 (18.4)	405 (20.0)	0.89 (0.63–1.26)	38 (63.2)	33 (42.4)	2.10 (0.79–5.58)	
Total	10 816 (16.2)	10 816 (15.0)	1.09 (1.02–1.18)	5389 (60.0)	5389 (57.4)	1.12 (1.04–1.21)	

**Table 3:** Relationship between passive smoking and stroke death in non-smoking and smoking population.

Stroke type	Non-smokers		Smokers		Total		
	Age-adjusted ORs (95% CI)	Fully adjusted ORs (95% CI)*	Age-adjusted ORs (95% CI)	Fully adjusted ORs (95% CI)^†^	Age-adjusted ORs (95% CI)	Fully adjusted ORs (95% CI)^‡^	
Hemorrhagic							
Men	1.10 (0.95–1.28)	1.20 (1.04–1.40)	0.97 (0.88–1.06)	1.05 (0.95–1.16)	1.02 (0.94–1.10)	1.09 (1.01–1.19)	
Women	1.15 (1.05–1.26)	1.14 (1.04–1.25)	1.31 (1.04–1.65)	1.23 (0.98–1.56)	1.17 (1.08–1.27)	1.16 (1.06–1.26)	
Total	1.12 (1.05–1.20)	1.13 (1.05–1.22)	1.00 (0.92–1.08)	1.07 (0.98–1.18)	1.06 (1.01–1.11)	1.10 (1.04–1.16)	
Ischemic							
Men	1.66 (1.36–2.02)	1.33 (1.09–1.64)	1.28 (1.12–1.47)	1.04 (0.90–1.20)	1.39 (1.24–1.55)	1.13 (1.00–1.27)	
Women	0.96 (0.84–1.10)	1.10 (0.95–1.26)	1.08 (0.78–1.51)	1.31 (0.92–1.87)	0.98 (0.86–1.11)	1.12 (0.98–1.27)	
Total	1.16 (1.04–1.28)	1.16 (1.03–1.30)	1.27 (1.12–1.43)	1.08 (0.95–1.24)	1.21 (1.12–1.30)	1.12 (1.03–1.23)	
All							
Men	1.23 (1.07–1.41)	1.25 (1.09–1.43)	1.04 (0.95–1.13)	1.04 (0.95–1.14)	1.09 (1.02–1.18)	1.10 (1.02–1.19)	
Women	1.10 (1.01–1.20)	1.13 (1.04–1.23)	1.25 (1.01–1.54)	1.25 (1.01–1.55)	1.12 (1.04–1.21)	1.15 (1.06–1.24)	
Total	1.13 (1.06–1.20)	1.14 (1.06–1.22)	1.05 (0.98–1.14)	1.07 (0.99–1.17)	1.10 (1.04–1.14)	1.10 (1.05–1.16)	

*Adjusted for gender, age, resident zones, ethnicity, and education; ^†^Adjusted for gender, age, resident zones, ethnicity, education, and years of active smoking; ^‡^Adjusted for gender, age, resident zones, ethnicity, education, and active smoking.

**Table 4:** ORs in different geographic zones of China.

	Foreland zone in north China	Foreland zone in south China	Inland zone in north China	Inland zone in south China
Hemorrhagic stroke	1.06 (0.94–1.20)	1.05 (0.95–1.16)	1.17 (1.01–1.35)	1.23 (1.09–1.40)
Ischemic stroke	1.23 (1.08–1.40)	1.17 (0.95–1.43)	1.11 (0.93–1.34)	0.87 (0.66–1.16)
All stroke	1.13 (1.02–1.25)	1.07 (0.97–1.17)	1.15 (1.01–1.31)	1.19 (1.06–1.35)

Liaoning, Beijing, Tianjin, Hebei, and Shandong belong to the northern coastal China, Jiangsu, Shanghai, Zhejiang, Fujian, Guangdong, and Guangxi belong to the southern coastal China, Helongjiang, Jilin, Inner Mongolia, Shanxi, Shaanxi, He’nan, Gansu, Ningxia, and Xinjiang belong to the northern inland China, and Hubei, Hunan, Anhui, Jiangxi, Chongqing, Sichuan, Guizhou, and Yunnan belong to the southern inland China in this study^26^. ORs were adjusted for gender, age, cities or rural counties, ethnicity, education, and active smoking.

**Table 5:** Effect of smoking doses on association between passive smoking and stroke death.

	Hemorrhagic stroke		Ischemic stroke		All stroke	
	Age-adjusted ORs (95% CI)	Fully adjusted ORs (95% CI)*	Age-adjusted ORs (95% CI)	Fully adjusted ORs (95% CI)*	Age-adjusted ORs (95% CI)	Fully adjusted ORs (95% CI)*
Diagnostic level 1						
Non-exposure	1.00	1.00	1.00	1.00	1.00	1.00
Exposure years of passive smoking						
1~19	1.09 (0.91–1.30)	1.09 (0.91–1.31)	1.28 (0.97–1.69)	1.26 (0.95–1.68)	1.13 (0.95–1.33)	1.14 (0.96–1.35)
20~39	1.03 (0.95–1.13)	1.11 (1.01–1.21)	1.27 (1.11–1.45)	1.20 (1.04–1.38)	1.08 (0.99–1.17)	1.13 (1.03–1.23)
40~	1.07 (0.99–1.15)	1.14 (1.04–1.24)	1.12 (1.00–1.26)	1.09 (0.95–1.24)	1.08 (1.00–1.16)	1.13 (1.04–1.22)
*p* for trend		0.001		0.023		<0.001
Cigarettes smoked daily by spouses						
1~9	0.91 (0.83–0.99)	1.00 (0.91–1.09)	1.21 (1.06–1.37)	1.06 (0.92–1.21)	0.97 (0.89–1.05)	1.02 (0.93–1.11)
10~19	1.02 (0.94–1.10)	1.11 (1.01–1.21)	1.29 (1.15–1.45)	1.21 (1.07–1.38)	1.07 (1.00–1.16)	1.13 (1.04–1.22)
20~	1.01 (0.93–1.10)	1.07 (0.97–1.18)	1.12 (0.98–1.27)	1.12 (0.97–1.29)	1.03 (0.95–1.11)	1.09 (1.00–1.19)
*p* for trend		0.001		0.010		<0.001
Diagnostic level 2						
Non-exposure	1.00	1.00	1.00	1.00	1.00	1.00
Exposure years of passive smoking						
1~19	1.03 (0.75–1.41)	0.98 (0.71–1.34)	0.76 (0.40–1.43)	0.55 (0.29–1.09)	0.98 (0.72–1.33)	0.89 (0.65–1.21)
20~39	1.15 (0.99–1.34)	1.10 (0.93–1.29)	1.31 (1.03–1.66)	1.07 (0.82–1.40)	1.18 (1.03–1.36)	1.09 (0.94–1.27)
40~	1.08 (0.96–1.21)	1.01 (0.88–1.16)	1.31 (1.10–1.56)	1.09 (0.88–1.35)	1.13 (1.01–1.27)	1.03 (0.90–1.17)
*p* for trend		0.259		0.116		0.191
Cigarettes smoked daily by spouses						
1~9	1.16 (1.02–1.32)	1.08 (0.93–1.26)	1.41 (1.17–1.72)	1.06 (0.84–1.32)	1.22 (1.08–1.38)	1.07 (0.93–1.22)
10~19	1.10 (0.97–1.23)	1.04 (0.91–1.19)	1.41 (1.19–1.68)	1.12 (0.91–1.37)	1.17 (1.05–1.30)	1.05 (0.93–1.19)
20~	1.09 (0.97–1.23)	1.04 (0.90–1.20)	1.30 (1.08–1.55)	1.07 (0.86–1.33)	1.14 (1.02–1.27)	1.04 (0.91–1.18)
*p* for trend		0.471		0.474		0.464
Total						
Non-exposure	1.00	1.00	1.00	1.00	1.00	1.00
Exposure years of passive smoking						
1~19	1.06 (0.91–1.24)	1.07 (0.92–1.26)	1.16 (0.90–1.50)	1.10 (0.84–1.42)	1.08 (0.94–1.26)	1.09 (0.94–1.26)
20~39	1.06 (0.98–1.14)	1.11 (1.02–1.20)	1.28 (1.14–1.44)	1.17 (1.04–1.33)	1.10 (1.03–1.18)	1.12 (1.04–1.20)
40~	1.07 (1.00–1.14)	1.11 (1.03–1.19)	1.18 (1.07–1.30)	1.10 (0.98–1.23)	1.09 (1.03–1.16)	1.11 (1.04–1.19)
*p* for trend		0.001		0.007		<0.001
Cigarettes smoked daily by spouses						
1~9	0.97 (0.90–1.05)	1.04 (0.96–1.12)	1.26 (1.13–1.40)	1.07 (0.95–1.20)	1.03 (0.97–1.11)	1.04 (0.97–1.12)
10~19	1.03 (0.97–1.10)	1.09 (1.01–1.17)	1.32 (1.20–1.45)	1.20 (1.08–1.34)	1.09 (1.03–1.16)	1.11 (1.04–1.19)
20~	1.03 (0.96–1.10)	1.07 (1.00–1.16)	1.17 (1.05–1.30)	1.12 (1.00–1.27)	1.06 (0.99–1.12)	1.08 (1.01–1.16)
*p* for trend		<0.001		0.023		<0.001

*Adjusted for gender, age, resident zones, ethnicity, education, and active smoking.

**Table 6:** Quantitative assessment of confounding bias in risk estimates of exposure vs. non-exposure and stroke.

Confounders	Relative risk between confounders and stroke	Prevalence of confounders	Prevalence of passive smoking	OR between exposure and confounders	“True” relative risk assumed	Apparent relative risk	% Bias*
Hypertension	4.1	0.19	0.30	1.01	1.00	1.0031	0.30
High TC	1.3	0.05	0.30	1.01	1.00	1.0001	0.01
Low HDL-C	2.9	0.34	0.30	1.01	1.00	1.0026	0.26
High TG	1.2	0.13	0.30	1.01	1.00	1.0002	0.02
Overweight	0.8	0.30	0.30	1.01	1.00	0.9996	−0.04
Sum of all positive biases							0.59

^*^[(Apparent relative risk - “True” relative risk)/“True” relative risk]*100; TC: total cholesterol; HDL-C: high density lipoprotein cholesterol; TG: triacylglyceride.
